# Oral versus topical maintenance antifungal therapy for recurrent vulvovaginal candidiasis: a systematic review and meta-analysis

**DOI:** 10.1186/s12905-026-04356-8

**Published:** 2026-03-18

**Authors:** Sara Ibrahim Abdelkader, Sally Aboelthana Aboelenin, Rahma Alaa Abdelhafez, Ashraf Fawzy Nabhan

**Affiliations:** 1 Faculty of Medicine, Galala University, Attaka, Suez Egypt; 2 Department of Obstetrics and Gynecology, Faculty of Medicine, Ain Shams University, Cairo, Egypt

**Keywords:** Vulvovaginal candidiasis, Antifungal agents, Systematic review

## Abstract

**Background:**

Recurrent vulvovaginal candidiasis is a substantial personal and healthcare burden. This systematic review assessed the effects of topical versus oral maintenance antifungal therapy in women with recurrent vulvovaginal candidiasis to improve outcomes.

**Methods:**

We conducted a comprehensive search of bibliographic databases (CENTRAL, MEDLINE/PubMed), citation indexes (Web of Science, Scopus), and clinical trial registries (ClinicalTrials.gov, WHO ICTRP) from inception to March 2025, without language or publication status restrictions. We screened reference lists of relevant studies and systematic reviews. We included randomized trials comparing topical versus oral maintenance antifungal therapy for recurrent vulvovaginal candidiasis. Three reviewers independently screened the records, assessed the full texts, extracted the data, and assessed the risk of bias. The primary outcome was the proportion of participants with no clinical or mycological recurrence at the end of follow-up. We performed a common-effect meta-analysis. We used the GRADE approach to assess the certainty of evidence and generate a summary of findings table.

**Results:**

We identified six eligible trials (1224 participants), five completed trials with results (792 participants) and one completed trial awaiting results (432 participants). All studies included women with recurrent vulvovaginal candidiasis. In the oral arm, five studies used fluconazole and one study used itraconazole. In the topical arm, three studies used clotrimazole, one used a combination of clotrimazole-diclofenac, one used fenticonazole, and one used nystatin. Five studies continued outcome measurement for six months after completion of the maintenance phase. Most of the included studies were judged to be at high risk of bias. The pooled estimates of the effects are not significantly different across all outcomes, including clinical or mycological recurrence, satisfaction, and adverse events. The evidence for all outcomes was very uncertain due to serious methodological concerns and imprecision.

**Conclusions:**

The evidence is very uncertain about the benefits and harms of oral versus topical antifungal maintenance therapy for women with recurrent vulvovaginal candidiasis. Given the substantial personal and healthcare burden of the condition, the uncertainty of the evidence leaves a crucial gap in guidance for clinicians and patients when choosing a maintenance strategy.

**Review registration:**

OSF osf.io/g7fzy.

## Background

Recurrent vulvovaginal candidiasis (RVVC) represents a significant chronic health condition, marked by numerous symptomatic episodes of vulvovaginal Candida infection occurring within a single year. This can negatively affect various aspects of life, including sexual relationships, psychological well-being, and overall self-esteem [1]. 

RVVC is defined in Europe as a minimum of four culture-confirmed symptomatic episodes of infection on an annual basis. In North America, RVVC is defined as the manifestation of three or more symptomatic infection episodes occurring within one year [2–6]. 

The precise burden of RVVC remains a challenge due to differences in case definition, inconsistencies in diagnostic criteria, and insufficient epidemiological research. Approximately 7% of women worldwide, equating to over 130 million women annually, are affected by RVVC. The annual economic impact of RVVC, attributable to the cost of treatment and diminished productivity among affected women, has been projected to reach approximately $14 billion [1, 7]. 

The comprehensive management strategy for RVVC encompasses mitigating exposure to risk factors, administering appropriate treatment for the initial Candida episode, and subsequently employing pharmacological agents to prevent recurrence [7]. Both topical and oral antifungal agents can control primary episodes of Candida infection; however, maintenance treatment regimens, which are administered either monthly or weekly for durations of up to six months, are sometimes required to control recurrence. It is frustrating to both women and caregivers that following the cessation of maintenance therapy, the recurrence rates among affected women can vary significantly, ranging from 50 to 80% [2, 5, 7, 8]. 

New oral agents, such as oteseconazole (a novel tetrazole) and ibrexafungerp (a first-in-class triterpenoid), have been approved [9] and a novel topical formula is currently in phase IIb/III trials [10], thereby expanding the pharmacological alternatives available for this condition. Currently, the effects of oral versus topical maintenance antifungal therapy are uncertain [11]. 

This synthesis of evidence assessed the benefits and harms of topical versus oral maintenance antifungal therapy in women with recurrent vulvovaginal candidiasis for improving outcomes.

## Methods

### Protocol and registration

We conducted this review following the methodological standards detailed in the *Cochrane Handbook for Systematic Reviews of Interventions* [12]. The prospectively registered protocol is available in the OSF repository [13]. The full review adhered to the preregistered protocol without deviations and was reported in accordance with the Preferred Reporting Items for Systematic Reviews and Meta-Analyses (PRISMA) guidelines [14]. 

### Eligibility criteria

We included published randomized controlled trials that compared oral versus topical maintenance antifungal agents in women with recurrent vulvovaginal candidiasis. RVVC was defined as a minimum of four culture-confirmed symptomatic episodes of infection or as the manifestation of three or more symptomatic infection episodes occurring within one year. We did not include cluster RCTs, crossover trials, quasi-randomized trials, nonrandomized trials, or observational studies. We did not include any retracted studies.

### Information sources

A comprehensive literature search was conducted on March 7, 2025. We searched bibliographic databases (Cochrane Central Register of Controlled Trials (CENTRAL), MEDLINE/PubMed), and citation indexes (Web of Science and Scopus). We searched clinical trial registries (ClinicalTrials.gov and the World Health Organization International Clinical Trials Registry Platform) to identify ongoing trials. We also searched reference lists and explored the cited-by logs of identified studies and previously published reviews. We did not apply any language or date restrictions.

### Search strategy

The search strategy was designed by the authors. We peer-reviewed the search strategy via the PRESS checklist [15]. We used the following search strategy for MEDLINE/PubMed: (“candidiasis, vulvovaginal”[MeSH Terms] OR ((candida[MeSH Terms] OR candidiasis[MeSH Terms] OR candida[Text Word] OR candidiasis[Text Word] OR monilia*[Text Word] OR thrush*[Text Word]) AND (“vaginal diseases”[MeSH Terms] OR “vulvar diseases”[MeSH Terms] OR vulv*[Text Word] OR vagin*[Text Word] OR vulvovagin*[Text Word] OR “vulvo vagin*”[Text Word] OR genit*[Text Word]))) AND (recurrence[MeSH Terms] OR (recur*[Text Word] OR relaps*[Text Word] OR refractory[Text Word])). We used the Cochrane highly sensitive search strategy for identifying randomized trials in MEDLINE: sensitivity- and precision-maximizing version (2008 revision) [16]. 

The detailed exact strategy adapted for each database is available in the OSF repository [13]. 

### Data management

#### Study selection

We imported the reports identified from the electronic search to the digital platform, Rayyan [17]. After the removal of duplicates, three authors (SAA, SIA, RAA) independently screened all the articles identified from the search in a double-blind manner. Each title was coded in Rayyan as “include”, “maybe”, or “exclude”. We retrieved and assessed the full texts of all the studies that were coded “include” or “maybe” during screening. Reporting the outcomes was not an eligibility criterion. Conflicts throughout the selection process were resolved by discussion. All decisions were adjudicated by the senior author (AN). Finally, we collated multiple reports of the same study so that each study, rather than each report, was the unit of interest in the review. We recorded the selection process in sufficient detail to complete a PRISMA flow diagram.

#### Data collection process

Three authors (SAA, SIA, RAA) working in pairs extracted data in duplicate via an electronic form that saved data to a spreadsheet. The senior review author (AN) checked the study characteristics for accuracy against the study report. All discrepancies were discussed in a daily meeting and corrections, if any, were made.

#### Data items

We extracted the study design, description of the included participants, description of the interventions in the trial arms of each study, outcomes, trial registration, funding sources, and publication metadata.

#### Outcomes

The primary outcome was the proportion of participants with no clinical or mycological recurrence at the end of follow-up. We also assessed the effects of interventions on the following outcomes: no clinical recurrence at the end of maintenance therapy, no clinical recurrence at the end of follow up, satisfaction, quality of life, and adverse events.

### Risk of bias in individual studies

Three authors (SAA, SIA, RAA) independently assessed the risk of bias in each trial via the Cochrane revised risk of bias tool 2 (RoB-2). RoB-2 is a “results-based” tool. Therefore, we assessed bias for a specific result reported in an individual study across the following domains (1) risk of bias arising from the randomization process; (2) risk of bias due to deviations from the intended interventions (effect of assignment to intervention); (3) risk of bias due to missing outcome data; (4) risk of bias in measurement of the outcome; and (5) risk of bias in selection of the reported result [18, 19]. 

Each domain was rated as “low risk of bias,” “some concerns,” or “high risk of bias.” An overall judgment of “low risk of bias” was assigned when all domains were rated as low risk. Trials were judged as having “some concerns” if one domain raised some concerns and none were rated as high risk. An overall “high risk of bias” judgment was assigned if at least one domain was rated as high risk, or if multiple domains raised some concerns that were considered to collectively represent a high risk of bias. We conducted a discrepancy check via the RoB-2 Excel tool and resolved any differences through discussion. If a consensus could not be reached, the senior author (AN) made the final decision.

### Synthesis

#### Summary measures

For dichotomous outcomes, we planned to calculate the RR via the inverse variance method for each study, which was then pooled. For continuous outcomes, we planned to pool the MDs between the treatment arms at the end of follow-up if all trials measured the outcome on the same scale; otherwise, we pooled the SMDs.

#### Unit of analysis issues

The unit of analysis was the participant for all included studies. For multiarm studies, we combined all relevant oral intervention groups in the trial into a single group and all relevant topical intervention groups into a single control group for relevant outcomes. We combined both the sample sizes and the number of participants with events from all groups for dichotomous outcomes. In case of a continuous outcome, we planned to combine means and standard deviations as per the recommended Cochrane methods [20]. We planned to exclude any irrelevant arm from the analysis.

#### Dealing with missing data

For all outcomes, we used an intention-to-treat to synthesize results [21]. As part of the risk of bias assessment, we assessed the levels of attrition in the included studies [21]. 

#### Assessment of heterogeneity

We planned to assess the clinical and methodological diversity of the included studies. To estimate statistical heterogeneity, we calculated the Chi² statistic, the between-study variance (Tau^2^), and the proportion of that variance not due to sampling error (I²). We interpreted an I² statistic estimate of 50% or greater accompanied by a statistically significant Chi² statistic (*P* < 0.1) as evidence of substantial heterogeneity. If we identified substantial heterogeneity, we planned to report it and investigate possible causes by following the recommendations in Sect. 10.10 of the *Cochrane Handbook* [21]. 

#### Synthesis of results

We used the common effect model to pool data from trials that were judged to be sufficiently similar. In the case of substantial statistical heterogeneity, we planned to explore the possible causes and to conduct a random-effects meta-analysis only if an average treatment effect across trials was considered meaningful [21]. 

We planned to conduct subgroup analysis to explore the following subgroups: diabetic versus nondiabetic women and albicans versus non-albicans candida species. We planned to assess subgroup differences by interaction tests available within R.

We planned to perform sensitivity analyses to explore the robustness of the pooled estimate using random effect model and fixed effect model.

The meta-analysis was conducted via R version 4 [22] and the package meta version 8 [23]. 

### Reporting bias assessment

We planned to assess nonreporting (including publication) bias by visually assessing funnel plot asymmetry, and by using Egger’s test in meta-analyses if data from at least 10 trials were available [24, 25]. 

### Certainty assessment

We assessed the certainty of evidence for each outcome by adopting the GRADE approach (Grading of Recommendations, Assessment, Development and Evaluation) [26, 27]. Study limitations, imprecision, consistency, indirectness, and publication bias form the basis of the assessment. We used GRADEpro GDT software [28] to produce a summary of findings table. All authors discussed and approved the assessments and decisions on downgrading the certainty of evidence.

### Patient and public involvement

No patients or members of the public were involved directly in the design of this study. Although not explicitly part of this project, the research question of this manuscript was inspired by the daily discussion between patients and doctors in our outpatient gynecologic clinics.

## Results

### Study selection

**Fig. 1:**
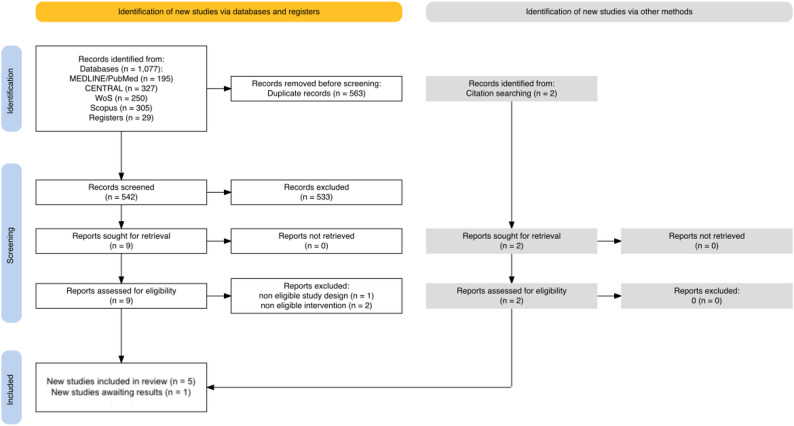
PRISMA 2020 flow diagram of study selection process

The search yielded 1109 potential records from electronic databases, registries and citation searches. After manual removal of duplicates, 542 titles and abstracts remained for screening. After screening, we retrieved 11 full text reports for assessment. We excluded three reports, one quasi-randomized study and two studies with ineligible interventions. The detailed selection process is depicted in the PRISMA flowchart, Fig. 1 [29]. 

We identified six eligible trials (1224 participants). Five completed trials [30–33, 35] with results of 792 participants, were included in the qualitative and quantitative synthesis. One multi-country multicenter completed trial [34] that recruited 432 participants from 29 centers is still awaiting results and was not included in the quantitative synthesis, Table 1.

**Table 1:** Characteristics of eligible studies

Study ID	Design	Country (number of sites)	Enrolled	Oral route	Vaginal route	Main findings
Five Eligible studies that were included in the quantitative synthesis						
Dobrokhotova 2024 [30]	Parallel	Russia (1)	206	Initiation: Fluconazole 150 mg orally every 72 h (3 doses in total on days 1, 4, and 7). Maintenance: Fluconazole 150 mg orally once a week for 3 months. After completion of treatment, observation continued for 6 months.	Initiation: Fenticonazole 600 mg topically twice at 72 h intervals Maintenance: Fenticonazole 600 mg once every 10 days for 3 months OR 600 mg twice at 72 h intervals once a month for 3 months OR 600 mg once every 10 days for 3 months followed by two courses of PRP-therapy. After completion of treatment, observation continued for 6 months.	At 6 months after completing maintenance therapy, recurrences occurred in 34.3% (33/96) of the main group and 46.4% (51/110) of the control group, respectively (*p* = 0.039).
Fan 2015 [31]	Parallel	China (1)	330	Initiation: Fluconazole 150 mg oral capsule on days 1,4 and 7 Maintenance: Fluconazole 150 mg orally once a week for 6 months. After completion of treatment, observation continued for 6 months.	Initiation: A dose of 20 MU *vaginal nystatin* daily for 14 days. Maintenance: 20 MU daily for 7 days before and after menstruation for 6 months. After completion of treatment, observation continued for 6 months.	Nystatin and fluconazole showed similar mycological cure rates after initial therapy 78.3% (119/152) and 73.8%(104/141) in the nystatin group and fluconazole group, respectively (95% CI, 0.749–2.197, p[0.05), during maintenance therapy 80.7% (96/119) and 72.7% (72/99) in the two groups, respectively (95% CI, 0.954–3.293, p[0.05), six months after stopping treatment 81.25% (78/96) and 82.19% (60/73) in the two groups, respectively (95% CI, 0.427–2.066, p[0.05), and RVVC caused by C. albicans 84.0% (89/106) and 81.8% (99/121) in the two groups, respectively.
Fardyazar 2007 [32]	Parallel	Iran (2)	124	Initiation: single oral dose of fluconazole capsule 150 mg. Maintenance: Fluconazole 150 mg weekly for 6 months, After completion of treatment, observation continued for 6 months.	Initiation: clotrimazole vaginal cream 5 g/day for 7 days. Maintenance: Clotrimazole 5 g twice a week for 6 months. After completion of treatment, observation continued for 6 months.	Recurrence rate at the end of 6-months follow up was 38.3% in the fluconazole group and 40% in the clotrimazole group (NS).
Fong 1992 [33]	Parallel	Canada (1)	44	Initiation: Itraconazole 200 mg orally daily (administered as 100 mg twice daily with meals) for 5 days. Maintenance: 200 mg twice weekly for 6 months After completion of treatment, observation continued for 6 months.	Initiation: Clotrimazole vaginal ovules 200 mg daily for 5 days, Maintenance: Clotrimazole 200 mg twice weekly for 6 months. After completion of treatment, observation continued for 6 months.	After the 6-months follow up period, recurrences of candida vaginitis were similar in both groups, 10 (47.6%) of patients on itraconazole (95% confidence interval (CI) 27–67%), versus 11 (64%) patients on clotrimazole (CI 41–87%), *p* = 0.15.
Shaheen 2020 [35]	Parallel	Egypt (1)	88	Initiation: Fluconazole 150 mg one oral capsule every third day for a total of three doses (days 1, 3, and 7). Maintenance: Fluconazole 150 mg one oral capsule every week for 6 weeks, then every 2 weeks for the remaining period of 3 months. One follow-up visit, 1 week after the end of maintenance treatment.	Initiation: Clotrimazole 100 mg vaginal tablet once daily at night for 14 days. Maintenance: Clotrimazole 100 mg vaginal tablet twice weekly for 3 months. One follow-up visit 1 week after the end of maintenance treatment.	A total clinical cure rate of 81% for fluconazole group and 76.1% for clotrimazole group. Total mycological cure rate was 80.5% for fluconazole and 77.3% for clotrimazole. There was no statistically significant difference regarding clinical or mycological cure rates in all visits between the two groups.
One completed study that was not included in the quantitative synthesis						
NCT04734405 [34]	Parallel, non-inferiority	Austria, Poland, Slovakia (35)	432	Initiation: Fluconazole 150 mg, one oral capsule on days 1, 4, and 7 and a daily dose of app. 5 g of placebo cream for 6 days (twice daily 2.5 g vulvar/ intravaginal application of cream), followed by 4 days of 2.5 g of placebo cream at bedtime. Maintenance: Fluconazole 150 mg, one oral capsule per week for 24 weeks and two doses of 2.5 g of placebo cream per week for 22 weeks (total of 44 single doses). After completion of treatment, observation continued for 6 months.	Initiation: app. 5 g of ProF-001 (Diclofenac-clotrimazole) for 6 days (twice daily app. 2.5 g vulvar/ intravaginal application of cream), followed by 4 days of app. 2.5 g of ProF-001 at bedtime and 1 placebo capsule on days 1, 4, and 7 Maintenance: 2 doses of app. 2.5 g of ProF-001 (Diclofenac-clotrimazole) per week for 22 weeks (total of 44 single doses) and 1 placebo capsule per week for 24 weeks. After completion of treatment, observation continued for 6 months.	The trial has been completed, and the final results have not yet been published.

### Study characteristics

We identified six eligible trials (1224 participants), five completed trials with results (792 participants) and one completed trial awaiting results (432 participants). Table 1 provides an overview of the key characteristics of all eligible studies [30–35]. All studies recruited participants in an outpatient setting. Two studies were conducted in Asia [31, 32], two studies in Europe [30, 34], one in North America [33], and one in North Africa [35].

All studies recruited participants with RVVC. Five studies [31–35] defined RVVC as four or more episodes of proven infection in the previous 12-month period. One study used 3 or more episodes per year for inclusion [30]. The predominant species found on culture at study entry was *Candida albicans*.

In the oral arm, five studies used fluconazole [30–32, 34, 35] and one study used itraconazole [33]. In the topical arm, three studies used clotrimazole [32, 33, 35], one used a combination of clotrimazole-diclofenac [34], one used fenticonazole [30], and one used nystatin [31].

Five studies continued outcome measurement for an observational period of six months after the completion of treatment [30–34]. Only one study had one final visit a week after the end of maintenance [35].

### Risk of bias

The assessment of the risk of bias for each of the predefined outcomes is described with each outcome. Briefly, in all the domains, the risks of bias for each outcome in the included studies were high or had some concerns. The overall risk of bias in all outcomes was judged to be high except for adverse events where two of the three included studies had some concerns and the third was judged to be at high risk of bias. Full assessments of the risk of bias, including judgments and justifications, are publicly available in the OSF repository [13]. 

### Results of syntheses

#### Participants with no clinical or mycological recurrence at the end of follow up

**Fig. 2:**
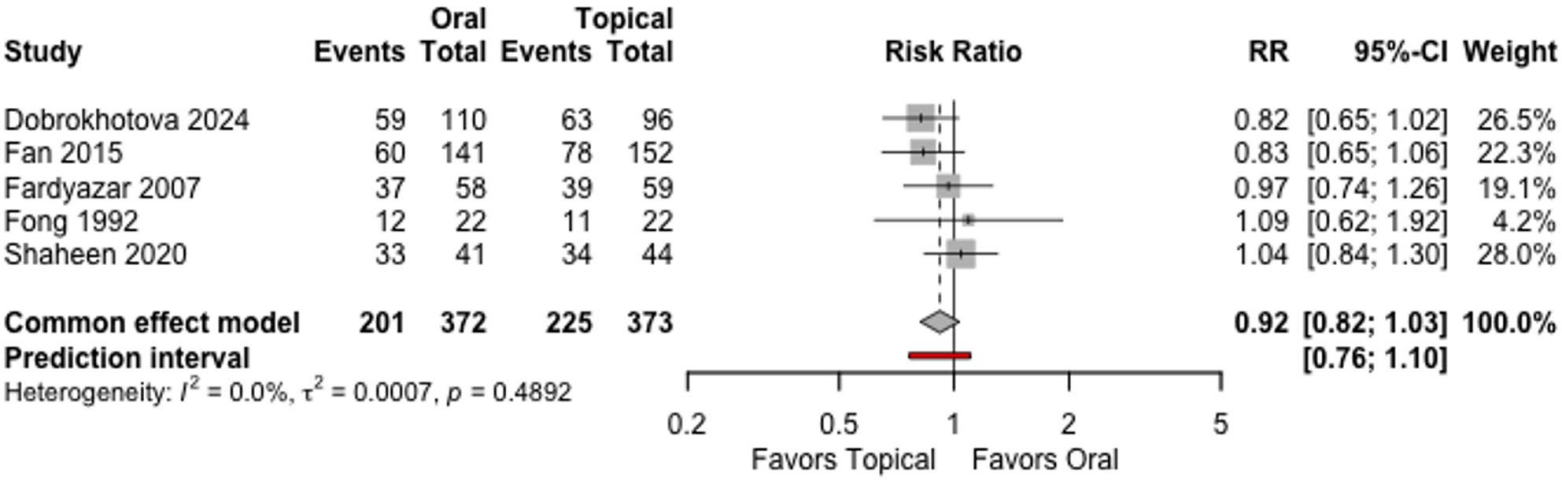
Participants with no clinical or mycological recurrence at the end of follow up

Five trials (745 women) reported participants with no clinical or mycological recurrence at the end of follow up. The risk ratio (RR) was 0.92 (95% confidence interval (CI) 0.82 to 1.03 under the common effect model, Fig. 2. We also calculated the prediction interval (95% PI is 0.76 to 1.10) to assess the range into which we can expect the effects of future studies to fall on the basis of the present evidence.

The effects of oral vs. topical antifungal treatment is very uncertain. We downgraded the certainty of the evidence by two levels due to very serious high risk of bias and one level for serious imprecision, Table 2.

**Table 2:** Summary of findings: Oral vs. topical maintenance antifungal therapy for women with recurrent vulvovaginal candidiasis

Outcome and follow-up	Patients (studies), *N*	Relative effect (95% CI)	Absolute effects (95% CI)			Certainty
			oral	topical	Difference	
Participants with no clinical or mycological recurrence at the end of follow up	745 (5 RCTs) [30, 31, 32, 33, 35]	RR = 0.92 (0.82 to 1.03)	540 per 1,000	497 per 1,000 (557 to 443)	43 fewer per 1,000 (from 97 fewer to 16 more)	⨁◯◯◯ Very low^a, b^
Participants with no clinical recurrence at the end of follow up	155 (2 RCTs) [32, 33]	RR = 1.02 (0.76 to 1.37)	544 per 1,000	555 per 1,000 (746 to 414)	11 more per 1,000 (from 131 fewer to 201 more)	⨁◯◯◯ Very low^a, b^
Participants with no clinical recurrence at the end of maintenance therapy	246 (3 RCTs) [32, 33, 35]	RR = 1.02 (0.95 to 1.09)	884 per 1,000	902 per 1,000 (964 to 840)	18 more per 1,000 (from 44 fewer to 80 more)	⨁◯◯◯ Very low^a, b^
Adverse events	539 (4 RCTs) [31, 32, 33, 35]	RR = 0.94 (0.41 to 2.18)	46 per 1,000	43 per 1,000 (100 to 19)	3 fewer per 1,000 (from 27 fewer to 54 more)	⨁◯◯◯ Very low^a, c^
Satisfaction	117 (1 RCT) [32]	RR = 0.54 (0.27 to 1.05)	172 per 1,000	93 per 1,000 (181 to 47)	79 fewer per 1,000 (from 126 fewer to 9 more)	⨁◯◯◯ Very low^a, b^

*CI* Confidence interval, *RR* Risk ratio

GRADE working group grades of evidence

High certainty: we are very confident that the true effect lies close to that of the estimate of the effect

Moderate certainty: we are moderately confident in the effect estimate: the true effect is likely to be close to the estimate of the effect, but there is a possibility that it is substantially different

Low certainty: our confidence in the effect estimate is limited: the true effect may be substantially different from the estimate of the effect

Very low certainty: we have very little confidence in the effect estimate: the true effect is likely to be substantially different from the estimate of effect

Explanations

^a^We downgraded the certainty of the evidence by two levels due to very serious risk of bias: Most of the included data contributing to this outcome come from studies with a high overall risk of bias

^b^We downgraded the certainty of the evidence by one level for serious imprecision: The optimal information size criterion was not met and the 95% CI includes no effect

^c^We downgraded the certainty of the evidence by three levels for extremely serious imprecision: The 95% confidence interval overlapped no effect and included both appreciable benefit and harm

#### Participants with no clinical recurrence at the end of follow up

**Fig. 3:**
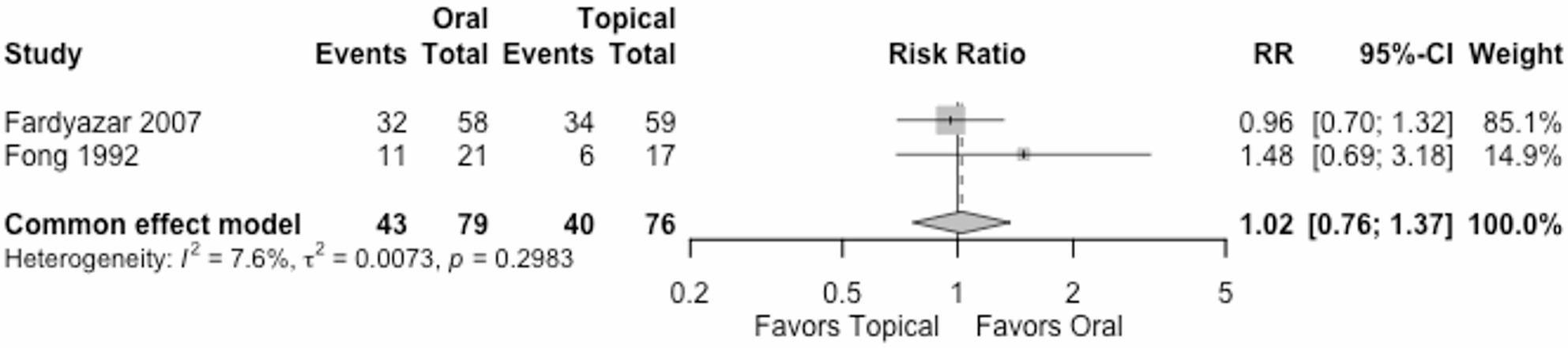
Participants with no clinical recurrence at the end of follow up

Two trials (155 women) reported participants with no clinical recurrence at the end of follow up. The RR was 1.02 (95% CI 0.76 to 1.37) under the common effect model, Fig. 3. The effect of oral vs. topical antifungal treatment is very uncertain. We downgraded the certainty of the evidence by two levels due to very serious high risk of bias and one level for serious imprecision, Table 2.

#### Participants with no clinical recurrence at the end of maintenance therapy

**Fig. 4:**
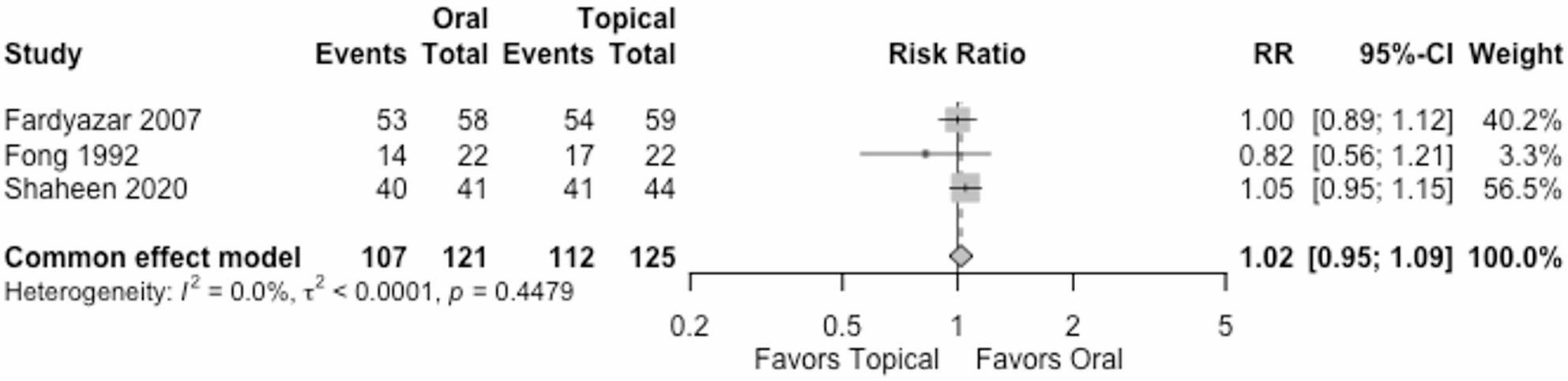
Participants with no clinical recurrence at the end of maintenance therapy

Three trials (246 women) reported participants with no clinical recurrence at the end of maintenance therapy. The RR was 1.02 (95% CI 0.95; 1.09) under the common effect model, Fig. 4. The effect of oral vs. topical antifungal treatment is very uncertain. We downgraded the certainty of the evidence by two levels due to very serious high risk of bias, and one level for serious imprecision, Table 2.

#### Adverse events

**Fig. 5:**
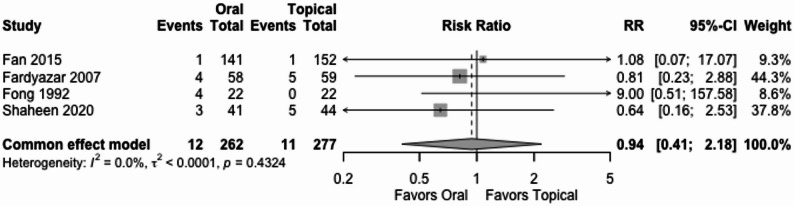
Adverse events

Four trials (539 women) reported participants with adverse events including vomiting and local side effects. The RR was 0.94 (95% CI 0.41 to 2.18) under common effect model, Fig. 5. The effect of oral vs. topical antifungal treatment is very uncertain. We downgraded the certainty of the evidence by three levels for extremely serious imprecision, and two levels due to very serious high risk of bias, Table 2.

#### Satisfaction

**Fig. 6:**
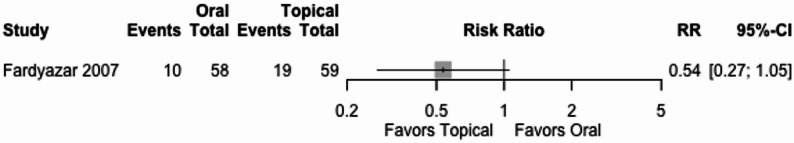
Satisfaction

One trial (117 women) reported participants with satisfaction. The RR was 0.54 (95% CI 0.27 to 1.05) under the common effect model, Fig. 6. The effect of oral vs. topical antifungal treatment is very uncertain. We downgraded the certainty of the evidence by two levels due to very serious high risk of bias and one level for serious imprecision, Table 2.

### Quality of life

None of the included studies reported a head-to-head comparison on the quality of life.

### Reporting biases

We included five studies, hence the assessment of publication bias using a funnel plot or standard tests was not possible. 

### Certainty of evidence

Compared with topical antifungal treatment, the effect of oral antifungal treatment is very uncertain about no clinical or mycological recurrence at the end of follow up, no clinical recurrence at the end of follow up, no clinical recurrence at the end of maintenance therapy, adverse events, and satisfaction.

We downgraded the certainty of evidence for all outcomes by two levels due to very serious high risk of bias. Studies contributing results to all outcomes were judged to have a high overall risk of bias.

For adverse events, we downgraded the certainty of the evidence three levels for extremely serious imprecision. The 95% CI is very wide and includes both appreciable benefit and harm. The two boundaries of the CI suggest very different inferences.

For all other outcomes (participants with no clinical or mycological recurrence at the end of follow up, participants with no clinical recurrence at the end of follow up, participants with no clinical recurrence at the end of maintenance therapy, and satisfaction), we downgraded the certainty of evidence by one level for serious imprecision because the CI includes no effect.

A summary of findings table presents the same information as the text above, with footnotes explaining judgments, Table 2.

## Discussion

Women affected by RVVC often endure persistent symptoms such as chronic itching, burning sensations, and reduced sexual activity, all of which contribute to a markedly diminished quality of life. RVVC presents therapeutic challenges due to limited treatment options, and selecting an appropriate maintenance regimen can be particularly complex. This systematic review assessed the benefits and harms of oral versus topical maintenance antifungal therapies for RVVC. We included data from five randomized controlled trials involving 792 participants. One completed trial is still awaiting results (432 participants). We found no significant differences between oral and topical treatments in terms of clinical or mycological recurrence, patient satisfaction, or adverse events. The certainty of the evidence for all outcomes was rated as very low.

Although a recent Bayesian network meta-analysis (NMA) identified a high risk of bias in most included studies, the authors still concluded that weekly maintenance therapy—whether oral (fluconazole, itraconazole, oteseconazole) or topical (clotrimazole)—was equally effective in preventing early RVVC recurrence [36]. In contrast, we consider the available evidence to be too uncertain to support conclusions of therapeutic equivalence. This position is consistent with a Cochrane review that, on the basis of four studies (543 participants) comparing oral versus topical antifungals, also judged the evidence to be inconclusive [11].

We did not find eligible studies comparing newer oral antifungals, such as oteseconazole and ibrexafungerp, with topical maintenance therapies. These new agents have not been directly compared even with other oral antifungal treatments.

Current evidence suggests that, in women with RVVC, both oral and topical antifungal therapies may be effective in reducing symptomatic recurrence compared with placebo or no treatment [11, 37]. We did not include studies comparing antifungal therapy to placebo or no treatment, because our objective was to determine the optimal route for maintenance therapy. Additionally, withholding effective treatment in this chronic condition may be ethically questionable.

While oral antifungal treatment probably improves mycological cure over topical treatment for uncomplicated vaginal candidiasis [38], this is not the case for maintenance therapy for RVVC. Healthcare providers must be aware of this important difference during the management of RVVC given its substantial personal and healthcare burden.

### Limitations of the evidence included in the review

The certainty of the evidence was consistently rated as very low for all critical outcomes due to serious methodological concerns and imprecision. Most of the included studies were judged to be at high risk of bias. The small number of trials reporting each outcome, coupled with relatively small sample sizes, led to wide confidence intervals. Only one study reported patient satisfaction, and few adequately captured long-term safety or quality-of-life measures, which are critical for chronic conditions such as RVVC.

### Limitations of the review processes used

This review might have limitations despite efforts to maintain methodological rigor. Although a comprehensive search strategy was conducted across multiple bibliographic databases and trial registries without restrictions on language or publication status, the possibility of missing relevant unpublished or nonindexed studies cannot be excluded. We could not assess the potential for publication bias by funnel plots or standard tests because of the small number of studies included in the main comparison of the review. One trial has been completed, and the results have not yet been published [34]. The absence of results from this large multi-country multicenter trial [34] regarding topical vs. oral antifungal maintenance therapy suggests a risk of reporting bias that could skew the available evidence.

The follow-up duration in one of the included studies [35] differs from that of the other included trials, as clearly reported in the study characteristics. Clinical heterogeneity—including variation in follow-up duration—does not justify exclusion when prespecified eligibility criteria are met, as post-hoc exclusion may introduce bias.

We could not conduct prespecified sensitivity and subgroup analyses because none of the included studies reported relevant stratified data.

### Implications for practice and future research

The additional contribution of our review stems from the fact that it reveals that current research evidence does not provide a reliable basis to recommend oral over topical maintenance antifungal therapy for recurrent vulvovaginal candidiasis, as the certainty of evidence is very low. For clinical practice, this uncertainty supports a shared decision-making approach that incorporates patient preferences, tolerability, cost, and availability rather than assuming therapeutic equivalence. From a guideline perspective, our findings underscore the need for cautious recommendations and highlight the urgent requirement for well-designed trials using standardized outcomes, including patient-reported measures, to inform future updates.

Future research should include newer oral antifungal agents, such as oteseconazole and ibrexafungerp, to expand therapeutic options in light of emerging resistance and treatment failure. Future trials comparing oral versus topical maintenance antifungal therapies, must be adequately powered, standardize the definitions of RVVC, use a core outcome set, and must focus on patient-reported outcomes, such as satisfaction and quality of life, which are particularly relevant for this recurrent condition.

## Conclusions

The evidence is very uncertain regarding the benefits and harms of oral versus topical antifungal maintenance therapy for women with recurrent vulvovaginal candidiasis. The uncertainty of the evidence leaves a crucial gap in guidance for clinicians and patients when choosing a maintenance strategy.

## Data Availability

The data, analysis script and materials supporting the conclusions of this article are available in the OSF repository, [10.17605/OSF.IO/G7FZY] . To facilitate reproducibility, this manuscript was written by interleaving regular prose and analysis codes.

## Abbreviations

CI: Confidence interval
GRADE: Grading of Recommendations, Assessment, Development and Evaluation
OSF: Open Science Framework
RoB: Risk of Bias
RR: Risk ratio
RVVC: Recurrent vulvovaginal candidiasis
